# Intermediate and low abundant protein analysis of vitamin D deficient obese and non-obese subjects by MALDI-profiling

**DOI:** 10.1038/s41598-017-13020-z

**Published:** 2017-10-03

**Authors:** Nasser M. Al-Daghri, Enrica Torretta, Daniele Capitanio, Chiara Fania, Franca Rosa Guerini, Shaun B. Sabico, Mario Clerici, Cecilia Gelfi

**Affiliations:** 1 Prince Mutaib Chair for Biomarkers of Osteoporosis, Biochemistry Department, College of Science, King Saud University, Riyadh, 11451 Saudi Arabia; 2 Department of Biomedical Sciences for Health, University of Milan, Segrate (Milan), Italy; 3 Clinical Proteomics Unit, Scientific Institute for Research, Hospitalization and Health Care (IRCCS) Policlinico San Donato, San Donato Milanese (Milan), Italy; 4 Don C. Gnocchi Foundation, IRCCS, Milan, Italy; 5 Department of Physiopathology and Transplants, University of Milan, Milan, Italy

## Abstract

Obesity is a pathological condition caused by genetic and environmental factors, including vitamin D deficiency, which increases the risk of developing cardiovascular disorders and diabetes. This case-control study was designed to verify whether serum profiles could be identified differentiating obese and non-obese Saudis characterized by vitamin D deficiency and pathological levels of triglycerides, high-density lipoprotein cholesterol and high total cholesterol levels. The serum protein profiles of 64 vitamin D deficient (serum 25(OH)D < 50nmol/L) individuals with metabolic syndrome and with (n = 31; BMI ≥ 30) or without (n = 33; BMI < 30) obesity were analyzed by a quantitative label-free mass spectrometry approach (MALDI-profiling), combined with different serum immunodepletion strategies (Human7 and Human14 immuno-chromatographies), to analyze the intermediate- and low-abundant protein components. The analysis of intermediate-abundant proteins (Human7) in obese *vs*. non-obese subjects identified 14 changed peaks (p < 0.05) in the m/z range 1500–35000. Furthermore, the Human14 depletion provided new profiles related to obesity (121 changed peaks). Among changed peaks, 11 were identified in the m/z range 1500–4000 Da by high-resolution tandem mass spectrometry, belonging to apolipoprotein CIII, apolipoprotein B100, alpha-1-antichymotrypsin and complement C3. Data herein show that distinct protein profiles identify specific peptides belonging to lipid metabolism and inflammation processes that are associated with obesity and vitamin D deficiency.

## Introduction

Obesity is a pathological condition caused by genetic and environmental factors. Given the number of possible complications, including cardiovascular diseases, dyslipidemia, and type 2 diabetes mellitus (T2DM), obesity is a diffused concern in modern society without distinction of ethnicity, gender, age, and socio-economic status. Due to increased food intake and sedentary lifestyle, it’s estimated that by 2030, the prevalence of obesity will reach 1.35 billion worldwide^1^. In particular, obesity is highly prevalent in the Middle Eastern region, at least in part because of the fast-paced industrialization and the consequent impact of improved welfare. The easy access to ready-made, fatty and salty foods is a central factor in this new lifestyle^2^. In 2014, the Saudi national survey on chronic diseases and their risk factors indicated that 28.7% of subjects were obese, with a significantly higher prevalence in women than men (33.5%)^3,4^. Clinically, obesity is defined as being grossly overweight which could potentially damage health, and is commonly quantified by the increment of the body-mass index (BMI), a standard parameter assessed considering the mass in kilograms divided by the square of the height in meters, which measures the presence of an excess of fat.

Another element associated with obesity is vitamin D deficiency^5^. Vitamin D is a steroid characterized by a hormone-like activity, able to regulate several genes involved in mechanisms associated with growth and development^6^. The major fraction of available vitamin D (~90%) comes from sunshine exposure. Concerning its deficiency, a theory has been proposed related to a lack of UV-B absorption causing a fall in serum 25-hydroxy-vitamin D (25(OH) D), and increment of fat mass and metabolic syndrome called “winter response”^7^. In the Middle East, even in the presence of abundant sunshine, severely low levels of serum 25(OH)D are observed^8^. The possible causes rely on the limited sun exposure, due to cultural habits, darker skin, limited outdoor activities, diet, lack of rules regulating vitamin D fortification of food^9^, and genetic predisposition^10^. A recent study showed that specific vitamin D receptor polymorphisms correlate with the onset of obesity and higher BMI^10^. Interestingly, these data underline the heterogeneity of obesity, a condition in which genetic variations can affect inflammosome-related genes, leading to an increased production of pro-inflammatory cytokines. In particular, it was shown that the up-regulation of these genes leads to severe forms of obesity which are associated with a considerable degree of inflammation.

Beside genetic studies, changes in protein expression in obese subjects and animal models have been conducted by proteomics approaches in biological fluids and tissues^11–13^. Serum circulating proteins were determined adopting a protein digestion prior liquid chromatography coupled to tandem mass spectrometry (LC–MS/MS) analyses^14–16^. This approach, despite being characterized by high-throughput and sensitivity provided by MS instruments, may undergo confounding effects related to the protein digestion step^13^.

Matrix-assisted laser desorption/ionization time of flight (MALDI-ToF) MS is a technology used for protein-expression profiling adopted in the present study^17–19^. This technique not only allows the detection of differently expressed peptides/small proteins, but also permits the analysis and grouping of a large number of samples, as required by clinical studies, thus assuring correct statistical power^20,21^.

Human serum represents an informative biological material for disease diagnosis, although the wide dynamic range of protein concentration hampers the discovery of pathophysiological mechanisms and the development of diagnostic assays, as the complexity of the plasma proteome exceeds the analytical capacity of MS approaches. The characterization of the human serum, in fact, is a demanding task, as protein concentration spans more than 10 orders of magnitude^22^. Moreover, the conventional shotgun proteomic approaches lack to detect and quantify proteins whose concentration is two to three orders of magnitude lower than that of the most abundant species^23^. From our experience, an effective strategy to overcome this issue is to remove up to 99% of the diagnostically uninformative proteins in order to analyze the low-abundant protein fraction of serum proteome and identify protein species and proteoforms directly related to disease pathogenesis at pg/mL level^20,24^. However, intermediate-abundant serum proteins can provide hints for a better understanding of alterations occurring in this range of protein concentration (µg/mL - ng/mL).

In this context, the present study was designed to verify whether distinct serum profiles could be identified that differentiate obese (BMI > 30) and non-obese (BMI < 30) Saudis. All subjects were characterized by vitamin D deficiency (serum 25(OH)D < 50 nmol/L) and pathological levels of circulating triglycerides (TG), high density lipoprotein cholesterol (HDL-C) and high total cholesterol levels (CHOL). Based on the subjects’ metabolic profile, we expect to identify putative biomarkers of obesity by adopting a quantitative mass spectrometry approach of intact proteins, named MALDI-profiling. This technique provides the differential abundance of intact endogenous circulating peptides^13^, coupled to a high resolution tandem MS (HR-MS/MS) for peptide identification. The peptides we identified belong to lipid metabolism and inflammation, and their dysregulation was indirectly confirmed by immunoblotting.

## Results

### Biochemical parameters levels assessment

Both obese and non-obese subjects were characterized by pathological levels of TG (>2.2 nmol/L) and HDL-C (<1.03 nmol/L), and all had low levels of vitamin D (<50 nmol/L). Furthermore, within each BMI (≥30 and <30), subjects were characterized by heterogeneous levels of LDL-C, and total cholesterol. These two parameters allowed to sub-group subjects based on LDL-C or CHOL. In particular, subjects were sub-grouped according to CHOL levels in “desirable” (<5.2 nmol/L), “borderline” (5.2–6.2 nmol/L), and “high” (>6.2 nmol/L). All subjects were characterized by low HDL-C and high TG levels, whereas LDL-C levels followed the CHOL trend. It should be also considered that the “high” CHOL category of non-obese subjects was characterized by higher CHOL levels compared to obese subjects (p < 0.05).

**Figure 1:**
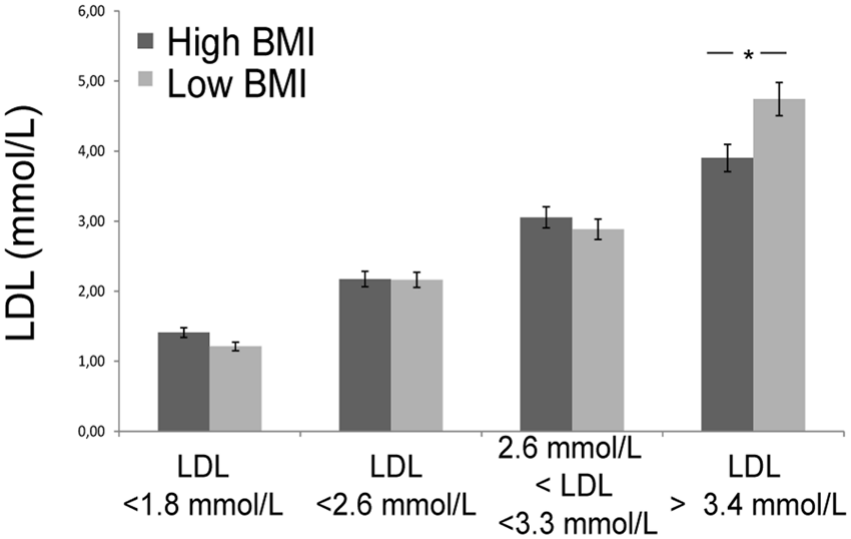
LDL-C samples classification. Sera were grouped according to LDL-C levels both for high BMI and low BMI. *Significant differences (Student’s t-test, p < 0.05) of high BMI versus low BMI.

Concerning the classification based on LDL-C and BMI, subjects could be grouped in 6 sub-groups as indicated in Fig. 1. Statically significant differences were observed for LDL-C > 3.4 mmol/L in obese *vs*. non obese subjects.

### MALDI-profiling analysis

To identify putative molecules characterizing obese subjects we employed a two-step approach: in a first phase, the 6 most abundant serum proteins were depleted; in the second step, 13 abundant proteins were depleted using commercially available columns. Profiling of intermediate- and low-abundant fractions was then performed on these samples to characterize the protein profiles related to obesity and vitamin D deficiency comorbidity. Multiple fractionations coupled to an high resolution tandem mass spectrometry (HR-MS/MS) analysis were adopted to identify the peptides in the range of 1500–4000 Da that were associated to the dysregulated species highlighted by MALDI-profiling.

Sera from 31 obese subjects and 33 non-obese subjects were analyzed to determine differences in scarcely-abundant protein profile, after Human 7 immunoprecipitation, and in low-abundant low molecular mass protein profile, after Human 14 immunoprecipitation.

**Figure 2:**
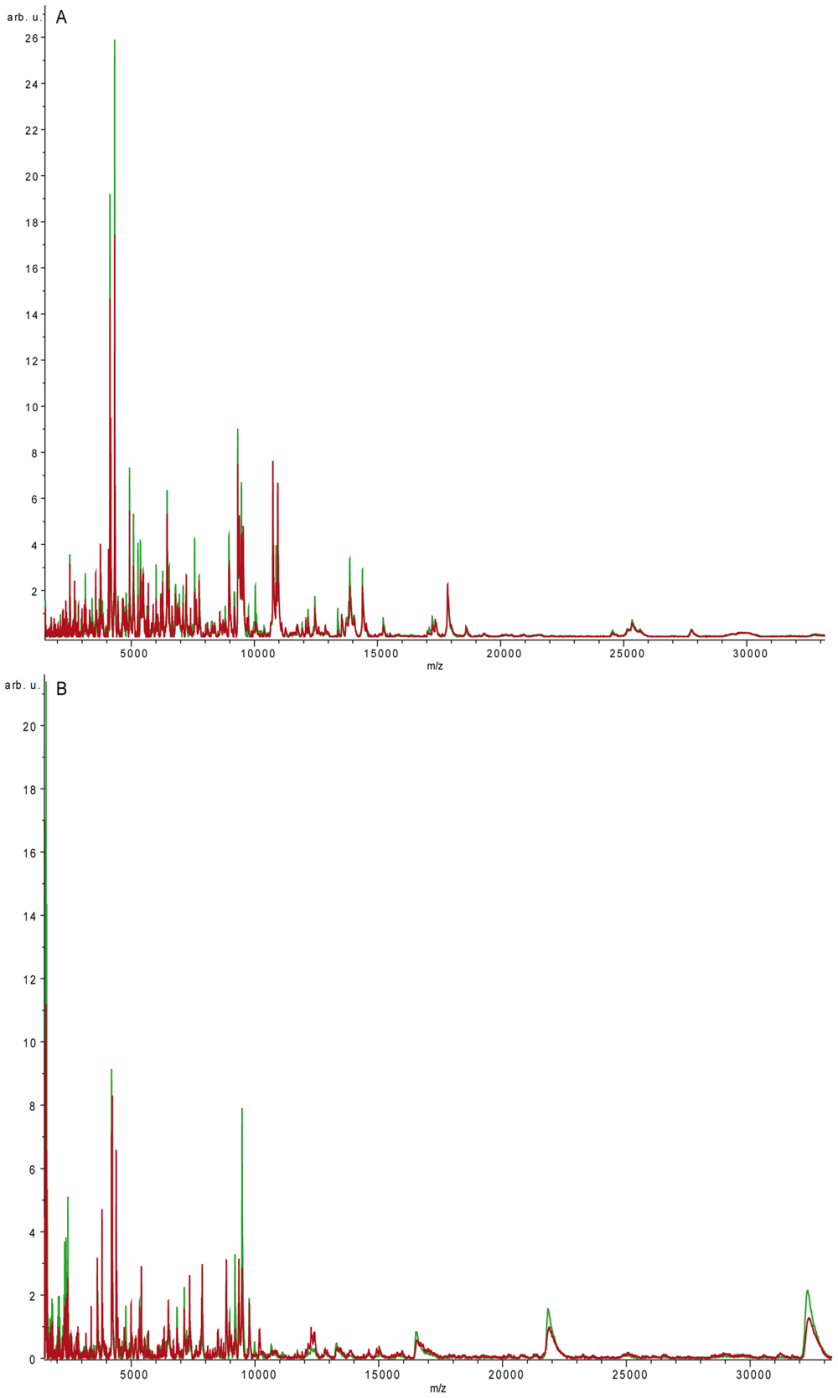
(**A**) MALDI-profiling analysis after Human7 depletion. MALDI profiling spectrum of Hu7 depleted serum (m/z range 1500–35000) from obese (average spectrum, red line) and non-obese subjects (average spectrum, green line). After immunodepletion, samples were spotted onto the AnchorChip target using DHAP matrix as described in method section. (**B**) MALDI profiling analysis after Human14 depletion. MALDI profiling spectrum of Hu14 depleted serum (m/z range 1500–35000) from obese (average spectrum, red line) and non-obese subjects (average spectrum, green line). After immunodepletion, samples were spotted onto the AnchorChip target using DHAP matrix as described in method section.

Utilizing Human 7 column, serum samples were depleted from the 6 most abundant proteins and the unbound intermediate abundance protein components were analyzed by MALDI-MS in the mass range of 1500 to 35000 m/z. The majority of detected signals were in the 1500–8000 m/z range, with the most abundant peaks at 4131 and 4321 m/z (Fig. 2A, Table S1).

These spectra underwent statistical analysis by ClinProTools software, and results showed that, within the 300 different peaks that were detected, 14 were “best separating peaks” (p < 0.05, CV < 20%). A second analysis was conducted on the same sera depleted from the 13 most abundant proteins, and the protein components were profiled by MALDI-MS from 1500 to 35000 m/z.

Obtained spectra, underwent statistical analysis utilizing the ClinProTools software, as previously described. Comparing obese and non-obese subjects, a total of 300 signals were detected in the 1500–35000 m/z acquisition range. Among them, 121 were differentially expressed (p < 0.05, CV < 20%) (Fig. 2B, Table S2). Moreover, we observed that profiles after Hu14 depletion (121 best separating peaks), showed a higher number of differentially changed peaks compared to Hu7 (14 best separating peaks).

Overall, one of the differentially expressed peak (4672 m/z) was common to fractions immunodepleted by Human 7 (Hu7) and Human 14 (Hu 14) chromatographies.

### Protein identification by high resolution tandem mass spectrometry

**Figure 3:**
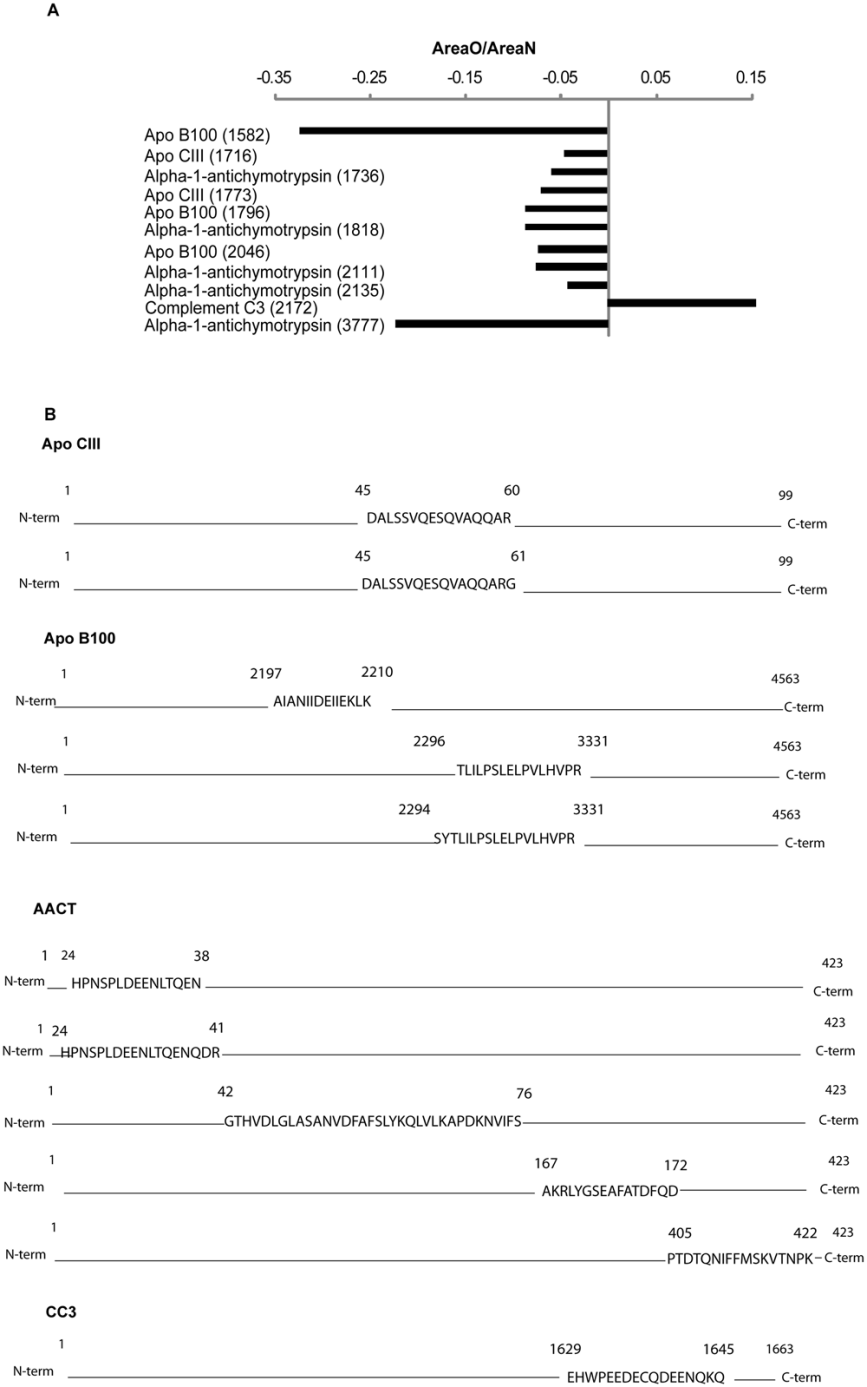
(**A**) Abundance data of identified “best separating” peptides. For each peptide, the m/z is indicated together with the abundance calculated as the ratio between the area observed in obese and that observed in non-obese subjects. (**B**) Identified peptides position within intact protein sequence for Apo CIII, Apo B100, AACT, and CC3 respectively.

In order to get better insight into the protein species that are dysregulated in obesity, the low and intermediate protein fractions were further investigated by performing an extensive high resolution MS/MS analysis coupled to a previous step of gel-filtration chromatography. In particular, protein species up to 4000 m/z were isolated from low and intermediate immunodepleted fractions, subjected to mass spectrometry analysis, and the list of identified peptides was compared with the list of dysregulated peaks provided by MALDI-profiling. Overall, 11 peptides corresponded to best separating peaks identified by MALDI-profiling (Fig. 3). In particular, 2 peptides belonged to apolipoprotein C III (Apo CIII, m/z 1716, and 1773 dysregulated in obesity after Hu14 depletion), five peptides to alpha-1-antichymotrypsin (AACT, m/z 1736, 1818, 2111, and 2135 dysregulated in obesity after Hu14 depletion and 3777 after Hu7), and three to apolipoprotein B100 (Apo B100, m/z 1582, 1796 and 2046 dysregulated in obesity after Hu14 depletion). These peptides were significantly less abundant in high BMI compared to low BMI subjects, whereas a peptide belonging to the complement cascade, complement C3 (CC3, m/z 2172 dysregulated in obesity after Hu7), was more abundant in high BMI compared to low BMI individuals (Fig. 3, panel A).

Sequence analysis of the identified proteins (Fig. 3 panel B) showed that peptides originate both from the N-terminal (1736, 2135, and 3777 peaks of alpha-1-antichymotrypsin), central (all Apo CIII and Apo B100 peptides; 1818 peak of AACT), and C-terminal regions (2111 peak of AACT; 2172 peptide of complement C3).

### Serum expression levels of lipid metabolism and inflammatory proteins

**Figure 4:**
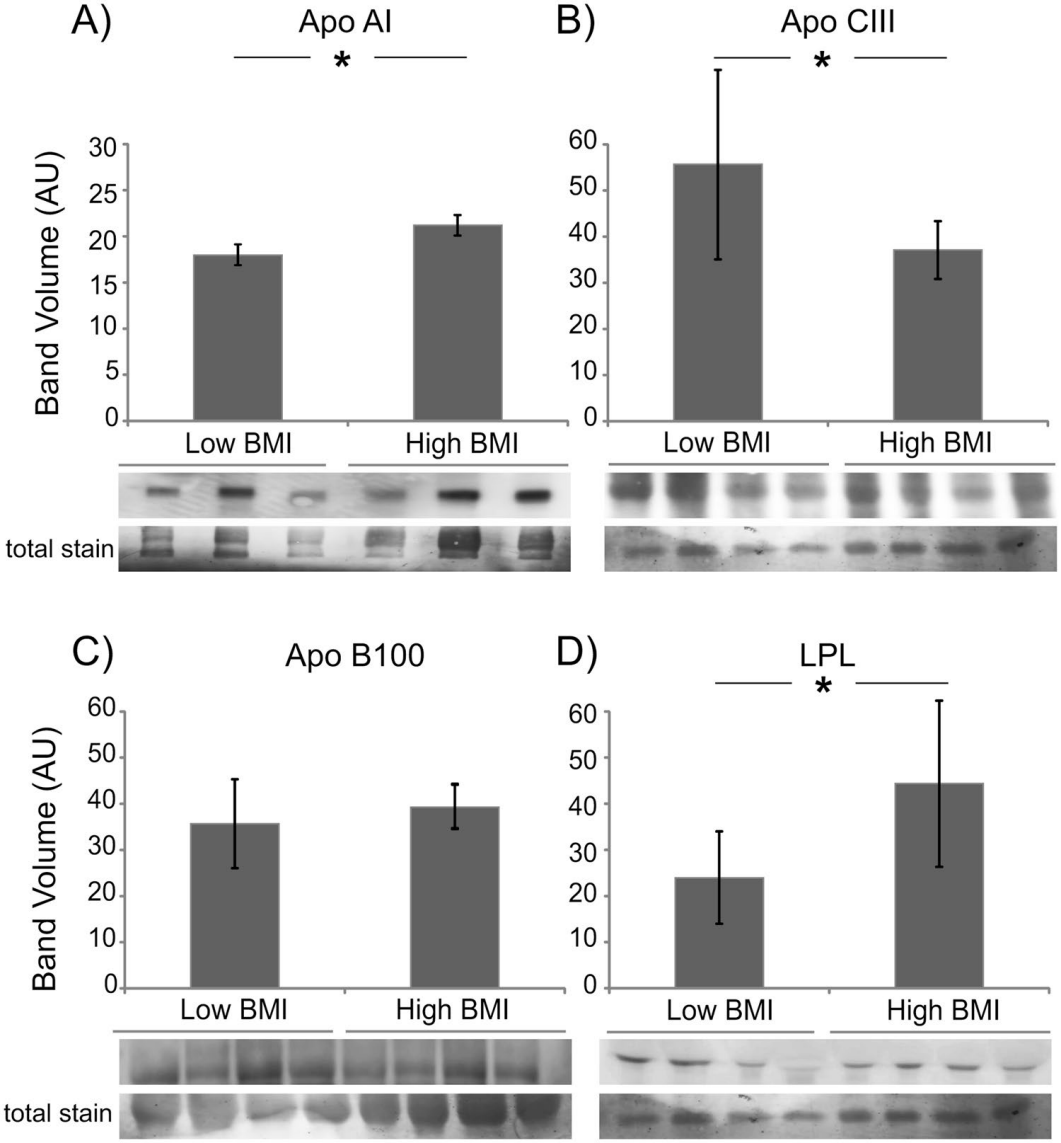
Expression analysis of proteins involved in lipid metabolism. Histograms (volumes normalized to total staining and reported as mean ± SD) and immunoblot closeups are shown for Apo A1, Apo CIII, Apo B100 and LPL, respectively. *Significant differences (Student’s t-test, p < 0.05) of high BMI versus low BMI.

The analysis of the peptides identified by MALDI suggested a dysregulation of three proteins involved in lipid metabolism and two proteins in inflammation. Some of these proteins were further investigated in sera from high and low BMI subjects by antigen-antibody reaction to validate results by an independent technology and to determine whether peptide dysregulation reflects changes of protein levels or processing associated to obesity. Thus, further analyses were performed on Apo A1, Apo CIII, Apo B100, and lipoprotein lipase (LPL) (Fig. 4). Apo A1 is the major component of HDL, plays a prominent role in the reverse transportation of cholesterol and is involved in anti-inflammatory and antioxidant processes^25^. Our results indicate Apo A1 intact protein levels were significantly increased (Student’s t-test, p < 0.05) in obese compared to non-obese (Fig. 4A) and proteolytic peptides levels appeared to be unchanged since they were not revealed by MALDI profiling.

Apo CIII intact protein was significantly under-expressed (p < 0.05) in obese following the same trend of related peptides (Fig. 4B). Conversely, Apo B100 and LPL levels were increased, in obese subjects, whereas proteolytic peptides decreased (Fig. 4C, D).

**Figure 5:**
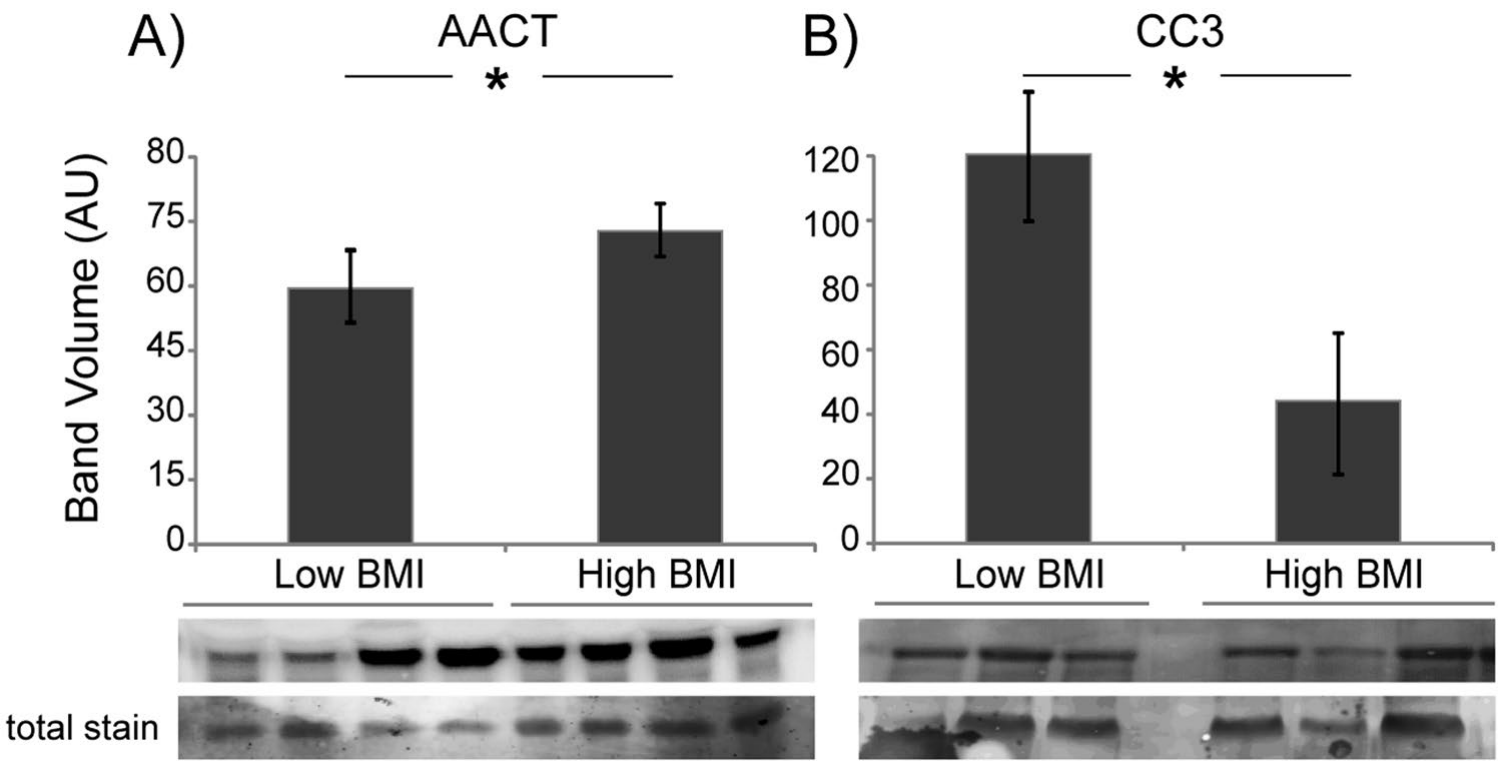
Expression analysis of proteins involved in inflammation. Histograms (volumes normalized to total staining and reported as mean ± SD) and immunoblot closeups are shown for AACT and CC3. *Significant differences (Student’s t-test, p < 0.05) of high BMI versus low BMI.

Finally, AACT and complement C3 were analyzed next to verify possible changes in these inflammation-related proteins; results are shown in Fig. 5.

Results showed that, as observed for Apo A1, Apo B100 and LPL, AACT intact protein was significantly overexpressed in obese subjects (Fig. 5A), whereas peptides identified by MALDI-profiling were less abundant in obese, suggesting that an altered protein processing is occurring.

Concerning CC3, in obese a decrement of the intact protein was detected (Fig. 5B), whereas peptides were more abundant.

## Discussion

Serum proteomics is a powerful approach focused on the assessment of circulating proteins/peptides, whose presence can be the result of genetic and/or metabolic changes. It is known, however, that serum protein species related to particular physio-pathological events are present in small concentration and, therefore, they remain undetectable unless high-abundant proteins are removed from sera prior to analyses. Along this line, serum immunodepletion followed by MALDI-profiling can be used as an initial screening to identify peptides or proteoforms associated to specific pathological conditions. Proteomics typically involves the use of endopeptidases, such as trypsin, to digest proteins into smaller peptide fragments of an ideal size for tandem MS. Such “bottom-up” proteomics strategies have been extensively used to analyze protein composition^14–16^. In contrast, the “top-down” proteomics approach used herein allows to precisely cluster subjects and to determine peaks statistically relevant to be candidate to the identification step, reducing the complexity of clinical studies and pinpointing the mass of peptides to be targeted for identification. In this context, data produced by MALDI-profiling are useful to prioritize molecules of interest to be targeted for identification and could allow the recognition of biomarkers for monitoring the disease onset and progression.

In the present study, we analyzed the protein profiles of a group of obese vitamin D deficient adults, comparing the results to those obtained in non-obese vitamin D deficient subjects; analyses were performed after intermediate and high abundant protein depletion. For the first time, these immunodepletions allowed us to assess the presence of peptides characterizing obesity, clearing the way to the identification of a set of putative biomarkers that could provide high diagnostic power.

The analysis of Human7 depleted fraction (intermediate-abundant proteins depletion) identified 14 separating peaks in the m/z range 1500–35000 comparing obese and non-obese subjects. This analysis indicated the existence of a specific protein pattern in obesity that became more evident by adopting the Human14 strategy. In this case, 121 changed peaks were detected.

Once serum has been extensively fractionated (*i.e*. immunodepletions, reverse phase chromatography and gel-filtration), HR-MS/MS analysis, as expected, identified protein species in the lipid metabolism and the inflammation pathways. Apo CIII and Apo B100 are important players of lipid metabolism, with 2 (m/z 1716, and 1773 less abundant in obesity) and 3 (m/z 1582, 1796 and 2046 less abundant in obesity) peptides, respectively being dysregulated after MALDI-profiling. In the first case, the expression of the intact Apo CIII showed the same trend of related peptides indicating that not only the endogenous peptide is decreased but also the intact protein. Apo CIII takes part in the formation of triglyceride-rich very low density lipoproteins and high density lipoproteins^26^, and modulates plasma TG levels^27^. Apo CIII inhibits LPL and dysregulation of this protein is associated to cardiovascular disease (CVD). LPL expression in serum was also investigated and, in agreement with lower Apo CIII levels in high BMI, resulted over-expressed in high BMI.

Peptides from Apo B100 were dysregulated as well in higher BMI individuals but the levels of the intact proteins, although augmented, were not significantly changed compared to the results obtained in low BMI subjects. In order to better define the serum signals related to the dysregulation of lipid metabolism, we also investigated the expression of Apo A1, the most abundant serum lipoproteins, involved in reverse and transport of cholesterol^25^. Interestingly, this protein was more abundant in high compared to low BMI individuals, confirming that lipid metabolism dysregulation is a hallmark of obesity^28^.

Obesity is also described as an energy imbalance pathology in which a smoldering degree of chronic inflammation promotes the development of insulin resistance and T2DM^29,30^. In the present study, five peptides of AACT were found to be less abundant (m/z 1736, 1818, 2111, 2135, and 3777), whereas a CC3 peptide (m/z 2173) was over expressed in obese subjects. The intact form of the first protein showed an expression at variance respect to peptides, suggesting a possible lower degradation among obese subjects. Unfortunately, although AACT is a known acute phase protein recently shown to be over-expressed in Alzheimer’s disease^31^, its role is still unclear. CC3, on the other hand, another acute-phase protein mainly produced by the liver, is one of the major serum proteins involved in the immune system complement pathway^32^. Moreover, it is known that the acylation stimulating protein (ASP), derived from CC3 cleavage, acts as an adipogenic hormone that stimulates triglyceride synthesis and glucose transport in adipocytes, regulating fat storage via activation of the PLC, MAPK and AKT signaling pathways^33,34^. CC3 protein was found to be reduced in the obese group, at variance with the peptide identified by MALDI-profiling. This observation seems to correlate the enhancement of CC3 processing to increased fat mass and weight gain and confirm that a chronic degree of inflammation, resulting in the activation of complement factors, is seen in obesity.

In conclusion, this study shows that specific protein profiles are associated with obesity, and provide identification of peptides in the range 1500–4000 Da. The abundance of peptides obtained after MALDI-profiling belonging to proteins involved in lipid metabolism and inflammation, was indirectly investigated by measuring the expression of the corresponding intact proteins by antigen-antibody reaction which confirmed a dysregulation of intact protein species and peptides from Apo A1, Apo B100, LPL, AACT whereas Apo CIII and CC3 behaved differently. The former is characterized by a decrement of both the intact protein and peptide, whereas the latter showed a decrement of the intact protein whereas the endogenous proteolytic product increased suggesting them as putative markers of an altered protein processing occurring in obesity. Notably, results of these analyses highlight that profile of peptides that characterizes obesity exists and can be identified, supporting the uniqueness of the study and the novelty of these results.

The identified peptides are circulating protein species generated by altered proteolysis in the bloodstream of precursor proteins and have the same abundance of their intact precursor, or be at variance. These findings suggest that a precise quantitation of specific endogenous peptides to monitor patients for CVD risk and complications can be obtained by MS through the combination between immunodepletion and MALDI profiling analysis, as highlighted in the comparison between high-BMI and low-BMI subjects, in which peptides of protein species related to lipid metabolism and inflammation pathways were dysregulated.

Compared to immunoassays, in which the quantitation of intact proteins could be misleading particularly when specific peptides abundance is at variance, such as in the case of AACT and CC3, MS offers more reliable data. The novelty of the approach we used is that it provided a set of peptides from Apo CIII, Apo A1, Apo B100, LPL, AACT, CC3 to be targeted in patients adopting MS methods based on absolute quantification through a single-reaction-monitoring (SRM) approach^35,36^, possibly allowing to identify disease specific patterns that can be translated to clinical laboratory for risk assessment in obesity and personalized medicine. However, this approach cannot be generalized since peptides/proteins require an extensive validation adopting automatized platforms for SRM and ELISA.

## Materials and Methods

### Participants and study design

Subjects enrolled in the study were all adult Saudis taken from the Vitamin D School Project Database of the Prince Mutaib Chair for Biomarkers of Osteoporosis, College of Science, King Saud University, Riyadh, Kingdom of Saudi Arabia^37^. By adopting the World Health Organization (WHO) criteria, BMI was used to classify subjects into two sub-groups: non-obese (BMI < 30 kg/m^2^) and obese (BMI ≥ 30 kg/m^2^). The present study conforms to the principles of Helsinki Declaration, and was approved by the Ethics Committee of the College of Science, King Saud University in Riyadh, Saudi Arabia (Ref No. 15/0502/IRB). All enrolled subjects provided their full informed consent.

Among the overall 64 samples selected, 31 were obese males and 33 non-obese male subjects as age-matched controls.

### Biochemical parameters

Vitamin D deficiency (serum 25(OH) D < 50nmol/L) was detected in all subjects (Table 1) as measured using electrochemiluminescence immunoassay on Roche Elecsys Cobas e411 analyzer (Roche Diagnostics, GmbH, Mannheim, Germany). Serum concentration of total cholesterol (CHOL), high density lipoprotein cholesterol (HDL–C), and triglycerides (TG) was evaluated using a chemical analyzer (Konelab, Espoo, Finland). Low density lipoprotein cholesterol (LDL-C) levels were indirectly calculated using the Friedewald’s formula.

**Table 1:** Participants’ characteristics.

	Obese subjects, N = 31		Non-Obese subjects﻿, N = 33	
Age, years (median, min-max, standard deviation)	44	(26–76) ± 13.77	45	(19–78) ± 17.2
BMI, kg/m^2^ (median, min-max, s.d.)	36.72	(33.3–47.88) ± 3.78*	23.39	(19.1–25.71) ± 1.63*
CHOL, mmol/L (median, min-max, s.d.)	5.40	(3.68–10.45) ± 0.98	4.86	(3.72–8.42) ± 1.62
HDL, mmol/L (median, min-max, s.d.)	0.78	(0.36–0.99) ± 0.15	0.67	(0.39–1.07) ± 0.15
LDL, mmol/L (median, min-max, s.d.)	3.15	(1.12–5.52) ± 0.9	2.36	(0.18–6.97) ± 1.37
TG, mmol/L (median, min-max, s.d.)	3.61	(2.66–15.36) ± 1.12	4.51	(2.71–7.33) ± 3.15
Vit D, nmol/L (median, min-max, s.d.)	32.91	(15.56–49.36) ± 8.76	32.12	(10.56–47.97) ± 10.9

*t-test p-value < 0.001.

### Sample preparation

For each sample, 25 μL of serum were diluted 1:4 with buffer A (Agilent Technologies, Santa Clara, CA), centrifuged with 0.22μm filters (Cellulose acetate Spin-X, Corning Inc., Bodenheim, Germany) to remove cells and debris, and injected onto an HPLC system (1200 series Agilent Technologies) equipped with immunodepletion columns. Two columns, MARS Human 7 (Hu7) and MARS Human 14 (Hu14) (Agilent Technologies), were used to selectively remove the most (from mol/L to mmol/L) and intermediate (from mol/L to nmol/L) abundant proteins. Specifically, Human 7 removes high abundant proteins by binding albumin, IgG, IgA, transferrin, haptoglobin, and antitrypsin, whereas Human 14 removes the high and intermediate abundant proteins by binding the six proteins indicated above plus alpha-2-macroglobulin, alpha-1-acid glycoprotein, IgM, apolipoprotein AI, apolipoprotein AII, complement C3 and transthyretin. These immunoaffinity steps provide the intermediate and low abundant protein fraction, respectively.

**Figure 6:**
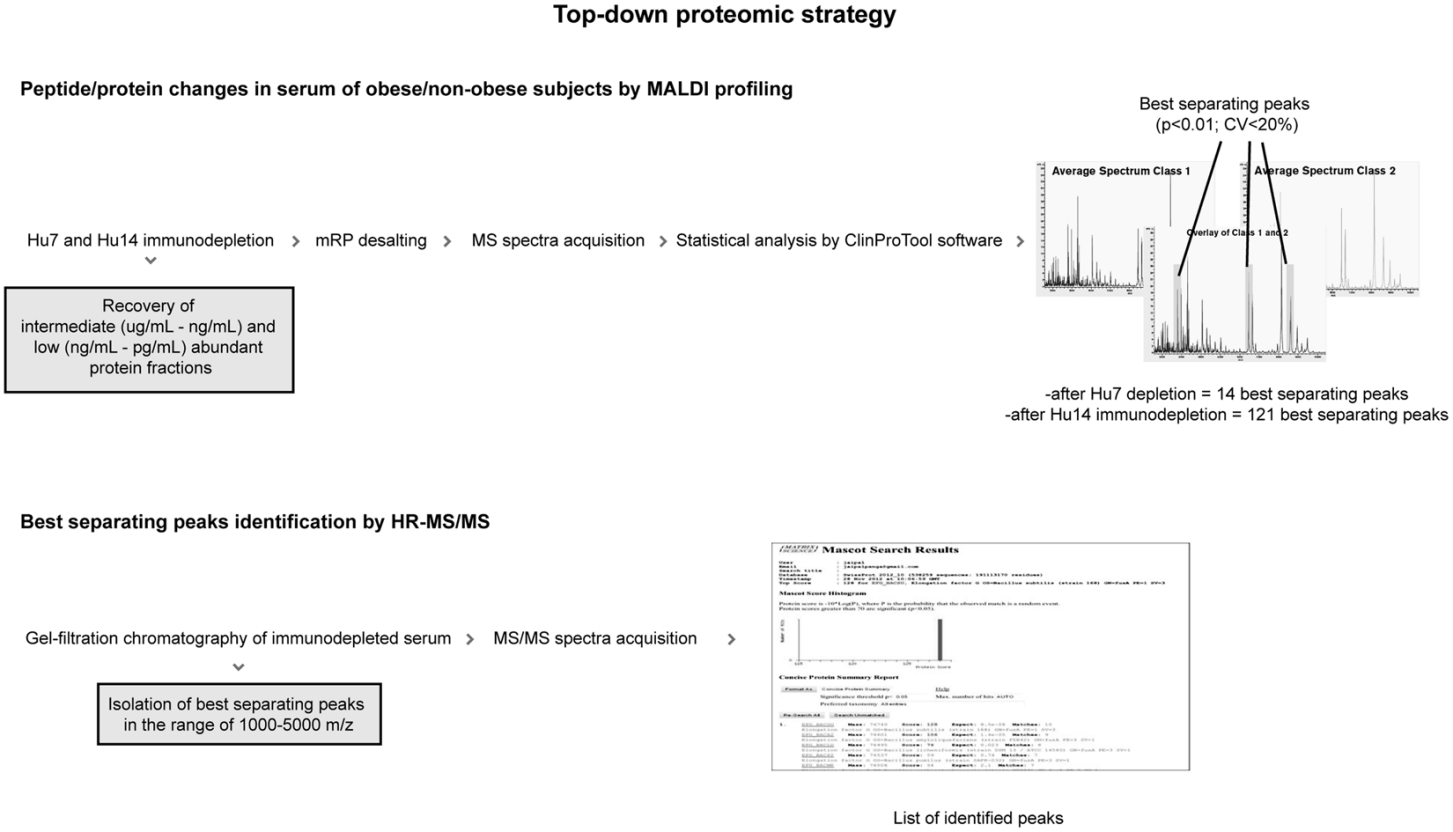
Diagram illustrating the strategy adopted for the identification of intact proteins/peptides circulating in the bloodstream, at variance in obese versus non-obese subjects.

Each fraction was desalted by reverse phase chromatography (mRP C18, Agilent Technologies), and quantified by BCA assay (Pierce, Rockford, IL) in order to normalize the loaded protein amount. Each immunodepleted fraction was then mixed with DHAP (dihydroxy-acetophenone) matrix, then spotted four times (one µg of protein content per spot) onto an AnchorChip target (Bruker Daltonics GmbH, Bremen, Germany), and analyzed by MALDI-ToF as previously described^24^ (Fig. 6A).

### MALDI-profiling

Spectra were acquired in linear positive modality (low resolution modality) using an Ultraflex III mass spectrometer equipped with Smartbeam laser, frequency of 100 Hz, Flex Control software v. 3.3 and Flex Analysis software v.3.3 (Bruker Daltonics). Spectrometer settings were: ion source 1, 25 kV; ion source 2, 23.5 kV; lens, 6.3 kV; deflection; mass suppression up to 800 m/z; pulsed ion extraction, 100 ns; detector gain voltage, 1798 V; electronic gain, 50 mV/full scale; sample rate, 1 GS/s; and laser attenuator offset, 80%. Spectra were collected using a fully automatic software, AutoXecute (Bruker Daltonics), whose parameters were: fuzzy control, off; laser power, 70%; total laser shots, 1000; random walk movement (25 shots per raster spot).

Raw mass spectra were analyzed by ClinProTools software v. 2.2 (Bruker Daltonics) using the following parameters: 800 resolution, Top Hat Baseline, 10% minimal baseline width, Savitsky–Golay smoothing. ClinProTools also allows to perform statistics which consisted in univariate analysis based on the evaluation of the abundance of each m/z signal among selected classes. In principle, each m/z signal represents a specific putative biomarker whose distribution shape must be evaluated in ClinProTools by Anderson-Darling test (AD test)^38^, which is a modification of the Kolmogorov-Smirnov (KS) test. If the p-value for the AD test is above 0.05 the Student’s t-test or ANOVA test must be considered; otherwise, the result from Wilcoxon/Kruskal-Wallis test has to be evaluated. Peaks generated by statistical analysis, from now named “best separating” peaks, were prioritized in obese and non-obese subjects, in particular only signals with Wilcoxon’s or Student’s t-test p-value < 0.05 and CV <20% were considered.

### Protein identification by high resolution tandem mass spectrometry

A pool of immunodepleted sera (Hu7 and Hu14) for obese and non-obese subjects was created and proteins were gel filtrated by BioSep SEC-S2000 column (Phenomenex Inc., Torrance, CA). Briefly, 120 micrograms of lyophilized immunodepleted sample after RP chromatography were loaded onto a BioSep SEC-S2000 column, and proteins separated with a 30 min isocratic flow (0.1% TFA and 40% ACN in water) at 1 mL/min. Thirty-second fractions were collected and individually checked by MALDI-MS to detect masses ranging from 1000 to 5000 Da following a top-down proteomic design based on direct ionization of intact molecules derived from endogenous proteolytic activity and released from tissues directly into the blood stream. The selected mass range contained some of the peaks identified as differentially expressed in MALDI profiling. These fractions were successfully analyzed by LC-MS/MS. Proteins were separated on an UltiMate3000 RSLCnano system (Dionex, Sunnyvale, CA) using a 200 mm × 75 μm id column (ProteoPrep C18 column, New Objective, Woburn, MA) as previously described^39^. The eluate was electrosprayed into an LTQ Orbitrap Velos operating in positive modality (Thermo Fisher Scientific, Madison, WI) through a Proxeon nanoelectrospray ion source^39^. Mass spectra were then analyzed using MaxQuant software v. 1.3.0.5 with an initial maximum allowed mass deviation set to 20 ppm for monoisotopic precursor ions and 0.5 Da for MS/MS peaks. Enzyme specificity was set as “none”, no missed cleavages were allowed, and N-terminal acetylation, methionine oxidation, and asparagine/glutamine deamidation were set as variable modifications. Raw spectra were converted in MGF files by Trans-Proteomic Pipeline software v. 5.0.0 (Institute for Systems Biology, Seattle, WA), and identified by correlation with uninterpreted spectra to *Homo sapiens* entries in NCBInr database using the on-line MASCOT software. The significance of the threshold was set at p-value < 0.05. Finally, the list of m/z of the identified peptides was compared with the list of MALDI changed peaks (Fig. 6B).

### Immunoblotting

Twenty-eight non-immunodepleted sera from obese males were randomly sub-pooled into 4 groups. The same was done with non-obese males. The sub-pooling was adopted as a method to reduce the variance among biological groups increasing the power to detect changes in expression above the average value of sub-pooled sample when few samples are available and the variance is high^40,41^. This approach allowed to obtain 4 biological replicates (composed by 7 samples for each pool) for both obese and non-obese subjects.

80 µg of serum proteins from each sub-pool were separated in duplicate by sodium dodecyl sulphate - polyacrylamide gel electrophoresis (SDS-PAGE), transferred and blocked onto a polyvinylidene fluoride (PVDF) membrane (300 mA; 180 min) utilizing a Transblot Cell from GE Healthcare (Uppsala, Sweden). The membranes were blocked overnight in tris-buffered saline (TBS) (20 mM Tris, 137 mM NaCl, 0.1% Tween, pH 7.5) containing 5% bovine serum albumin (Sigma Aldrich) and incubated with the following primary antibodies: anti-Apo A1 (Santa Cruz Biotechnology, Santa Cruz, CA, 1:400), anti-Apo CIII (Thermo Scientific, 1:2000), anti-Apo B100 (Thermo Scientific,1:1000), anti-LPL (Thermo Scientific, 0.48 mg/mL), anti-AACT (Thermo Scientific, 2 mg/mL), and anti-CC3 (Thermo Scientific, 1:1000). Horseradish peroxidase-conjugated anti-mouse (GE Healthcare, 1:4000) was used as secondary antibody for Apo A1; anti-goat (Santa Cruz Biotechnology) was used for Apo CIII, Apo B100 (dilution 1:5000) and CC3 (dilution 1:10000), whereas anti-rabbit (GE Healthcare,1:10000) was used for LPL and AACT.

Proteins were visualized by chemiluminescence (ECL Prime kit, GE Healthcare). Band intensities were detected using an Image Quant LAS 4000 mini imager (GE Healthcare) and assessed with the Image Quant TL 8.1 analysis software (GE Healthcare). The data were normalized against the total amount of proteins stained by Sypro Ruby and subjected to a Student’s t-test by comparing obese vs. non obese. Differences were considered significant at p < 0.05.

### Data Availability

The data generated during and/or analysed during the current study are included in this published article (and its Supplementary Information files). When needed, further information are available from the corresponding author on reasonable request.

## Electronic supplementary material

Supplementary Information
