# Supplementary material for: Intermediate and low abundant protein analysis of vitamin D deficient obese and non-obese subjects by MALDI-profiling
Source: Sci Rep. 2017 Oct 3;7:12633. doi: 10.1038/s41598-017-13020-z (PMC5626753; doi:10.1038/s41598-017-13020-z)
Supplement: Supplementary file 1 — Supplementary Information [file 41598_2017_13020_MOESM1_ESM.pdf]

Intermediate and low abundant protein analysis of vitamin D deficient obese and non-obese subjects by MALDI-profiling

Nasser M. Al-Daghri,[1] Enrica Torretta[2], Daniele Capitanio[2], Chiara Fania[3], Franca Rosa Guerini[4], Shaun B. Sabico[1], Mario Clerici[4,5], Cecilia Gelfi[2,3,*]

1 Prince Mutaib Chair for Biomarkers of Osteoporosis, Biochemistry Department, College of Science, King Saud University, Riyadh 11451, Saudi Arabia;

2 Department of Biomedical Sciences for Health, University of Milan, Segrate (Milan), Italy;

3 Clinical Proteomics Unit, Scientific Institute for Research, Hospitalization and Health Care (IRCCS) Policlinico San Donato, San Donato Milanese (Milan), Italy;

4 Don C. Gnocchi Foundation, IRCCS, Milan, Italy.

5 Department of Physiopathology and Transplants, University of Milan, Milan, Italy;

* cecilia.gelfi@unimi.it

Supplementary table S1. Differentially expressed peaks ($p<0.05$) obtained by comparing high and low BMI subjects after Hu7 immunodepletion. m/z, p-values of the Wilcoxon test, and averages areas for each dysregulated peak are indicated.

**m/z**	**p-value**	**High BMI Average expression**	**Low BMI Average expression**
2110	0.00621	25.28	21.74
2159	0.0488	24.28	20.92
2172	0.0131	30.82	25.88
2737	0.0435	34.20	40.39
3406	0.00151	46.14	59.42
3422	0.0144	39.40	46.86
3777	0.037	39.35	44.93
4672	0.0488	69.08	61.93
5107	0.0228	93.41	103.15
5271	0.0354	49.45	64.41
5292	0.00204	24.72	30.99
5487	0.0354	94.85	79.70
7285	0.0488	41.09	34.56
12875	0.000845	98.25	82.57

Supplementary table S2. Differentially expressed peaks ($p<0.05$) obtained by comparing high and low BMI subjects after Hu14 immunodepletion. m/z, p-values of the Wilcoxon test, and averages areas for each dysregulated peak are indicated.

m/z	p-value	High BMI Average expression	Low BMI Average expression
1530	0.00000555	107.81	181.11
1556	< 0.000001	104.57	210.67
1582	< 0.000001	78.49	165.00
1655	0.0345	46.79	43.30
1716	0.0303	34.73	38.59
1735	0.00213	38.80	44.53
1773	0.00168	54.44	64.13
1796	0.000406	55.96	68.33
1818	0.0000755	43.84	53.48
1913	0.00497	35.86	31.95
2046	0.00125	50.56	59.77
2059	0.0000117	44.52	57.96
2111	0.0000646	57.50	68.37
2135	0.0283	24.46	26.98
2325	0.00191	45.43	55.01
2692	0.00326	28.20	24.93
3219	0.0187	29.48	27.18
3358	0.000504	41.67	37.1
3700	0.000776	30.85	26.28
3861	0.0018	33.34	29.34
3874	0.029	18.94	17.38
3956	0.0038	46.50	42.16
3999	0.00416	24.49	22.77
4139	0.000534	27.00	22.76
4164	0.0015	32.78	29.91
4342	0.000064	37.25	31.60
4516	< 0.000001	71.84	59.33

4558	0.00000127	33.50	27.41
4581	0.00052	30.94	27.50
4672	0.00912	30.95	27.85
4711	0.0000109	57.33	49.1
4816	0.0333	26.41	28.87
5152	0.000881	36.56	32.79
5292	0.00734	37.19	33.72
5428	0.00855	40.72	38.23
5494	0.0419	26.91	24.32
5528	0.00356	34.80	29.84
5710	0.0000835	26.31	22.15
6140	0.0000117	21.37	17.84
6619	0.000881	26.82	23.42
6717	0.0457	24.90	22.91
7037	0.000136	30.50	26.49
7235	0.00391	38.25	51.89
7686	0.0271	26.04	23.58
7732	0.0125	22.09	19.77
7974	0.00297	54.56	47.69
8178	0.00213	28.06	24.99
8277	0.0338	27.18	25.40
8373	0.0128	17.71	16.31
8393	0.00326	45.01	41.58
8613	0.0000148	37.95	30.95
8631	0.0000616	25.92	21.31
8728	0.00000932	26.67	21.30
8936	< 0.000001	19.07	14.24
10856	0.002	34.33	30.02
10925	0.014	21.68	19.10
10951	0.0000467	29.92	25.66
11232	0.002	47.89	43.16
12049	0.000534	58.65	50.70
12752	0.00894	21.73	19.77

12893	0.0387	19.68	18.51
12910	0.000769	16.86	14.84
12984	0.00297	39.18	34.83
13292	0.0308	159.93	171.41
13412	0.000534	44.36	40.03
13463	0.000458	66.10	58.78
13562	0.00212	22.83	20.27
13726	0.000473	34.45	30.51
13807	0.0108	25.04	22.37
13834	0.00213	18.57	16.03
13865	0.0000688	18.97	15.94
14385	0.000064	42.70	36.19
14527	0.0277	34.34	31.51
14595	0.00628	99.58	90.24
14788	0.0093	36.68	32.94
15215	0.000072	48.20	41.24
15277	0.002	31.16	27.42
15537	0.000032	41.24	34.56
15942	0.0000143	49.03	39.97
16505	0.0000117	107.9	135.45
16904	0.00091	56.21	49.79
16990	0.00155	27.16	23.63
17113	0.00227	86.23	77.39
17224	0.0258	92.25	85.84
17368	0.00645	27.22	24.30
17993	0.000016	27.61	23.86
18046	0.000534	49.70	43.82
18415	0.0000328	48.31	41.29
19166	0.0022	42.82	37.63
19201	0.000939	31.54	27.74
20278	0.000101	71.11	59.61
21349	0.0000781	37.24	31.13
21393	0.0016	51.95	45.08

23233	0.0000148	73.90	62.00
25379	0.000109	55.23	47.15
25495	0.000185	66.57	57.13
25706	0.0000117	45.79	38.47
25773	0.0000117	33.79	28.14
25820	0.0000148	27.6	22.65
26099	0.0373	34.27	31.56
26268	0.00119	27.41	23.28
26317	0.0029	34.67	30.16
26510	0.00000475	72.93	59.25
26574	0.0000419	36.14	29.47
26637	0.0000634	42.45	35.42
26770	0.000348	54.91	47.25
27538	0.0000667	45.39	37.70
28481	0.000534	33.88	28.85
28567	0.000032	52.66	44.25
28852	0.000337	26.24	22.22
28967	0.002	55.64	48.07
29122	0.0000506	63.85	54.68
29283	0.0000549	39.20	33.72
29431	0.00453	32.95	29.03
29580	0.000337	49.73	43.13
29668	0.000545	30.55	26.23
29885	0.0000281	39.70	33.47
29942	0.000163	53.93	45.49
30110	0.00191	35.67	31.15
30566	0.0000581	57.87	47.89
31700	0.0000598	71.78	58.53

Supplementary Table S3. Peptide identification. Mass spectrometry data concerning proteins identified after by LC-MS/MS are shown together with protein Uniprot accession number.

Name	Uniporot AC	Measured m/z	Theroretical mass	Charge status	Calculated mass	D	Mascot Score	Peptide sequence
**Apolipoprotein CIII**	P02656	858,9406	1.715,8667	2	1715,8438	0,0228	27,54	DALSSVQESQVAQQAR
		887,4456	1.772,8766	2	1772,8653	0,0113	47,5	DALSSVQESQVAQQARG
**Apolioprotein B100**	P04114	899,0645	1.796,1145	2	1796,0924	0,0221	45,71	TLILPSLELPVLHVPR
		1.024,1138	2.046,2130	2	2046,1877	0,0253	40,85	SYTLILPSLELPVLHVPR
		528,3505	1.582,0297	3	1581,9341	0,0956	20,86	AIANIIDEIIEKLK
**Alpha-1-antichymotrypsin**	P01011	868,9025	1.735,7904	2	1735,7649	0,0255	26,95	HPNSPLDEENLTQEN
		606,9634	1.817,8683	3	1817,8584	0,0099	24,84	AKRLYGSEAFATDFQD
		712,6609	2.134,9610	3	2134,9515	0,0095	27,36	HPNSPLDEENLTQENQDR
		756,4139	3777,033	5	3777,0141	0,0189	20,01	GTHVDLGLASANVDFAFSLYKQLVLKAPDKNVIFS
		704,6918	2111,0537	3	2111,0358	0,0179	26,24	PTDTQNIFFMSKVTNPKQ
**Complement C3**	P01024	724,9554	2171,8443	3	2171,8338	0,0106	26,92	EHWPEEDECQDEENQKQ

Supplementary Data. Full-lenght blot images.

A) Apo CIII

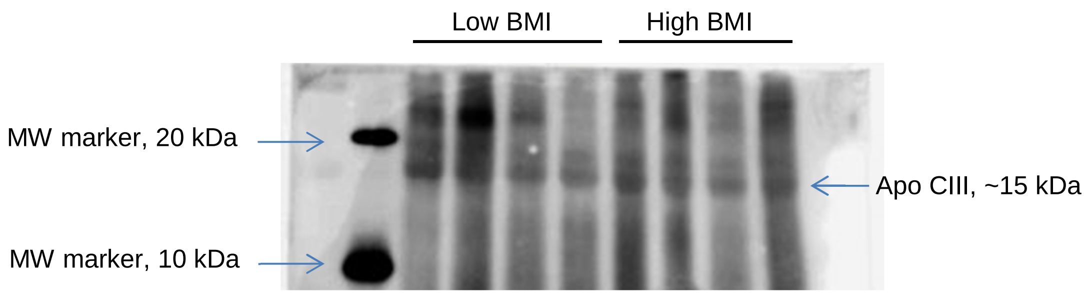

B) LPL

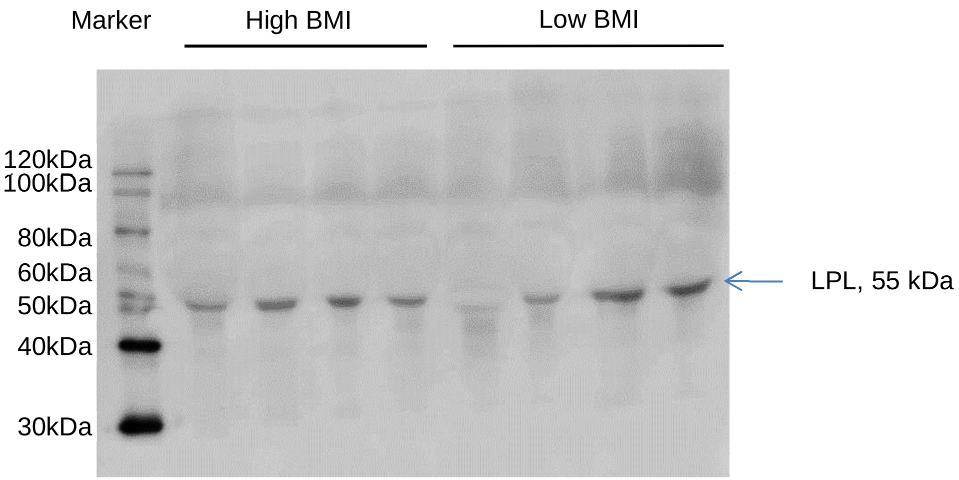

C) Apo B100

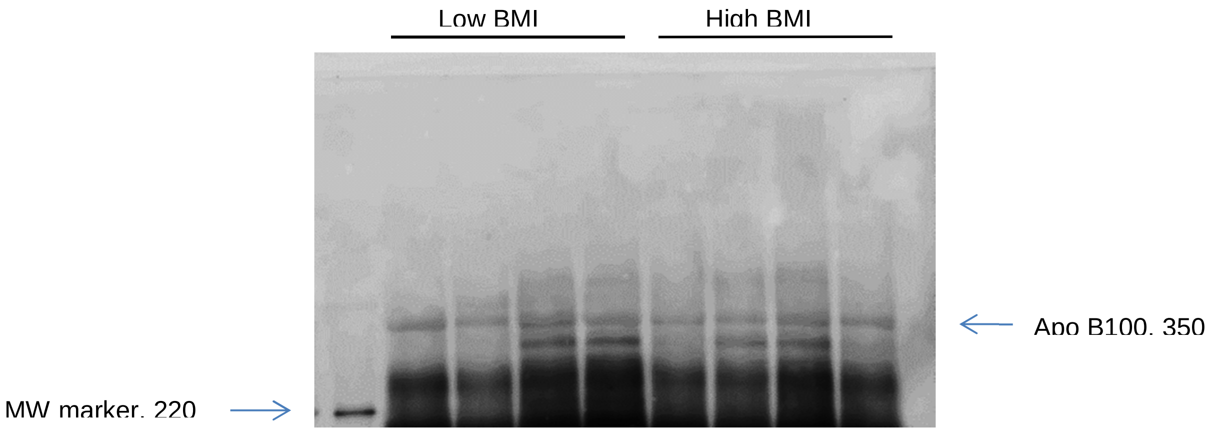

D) AACT

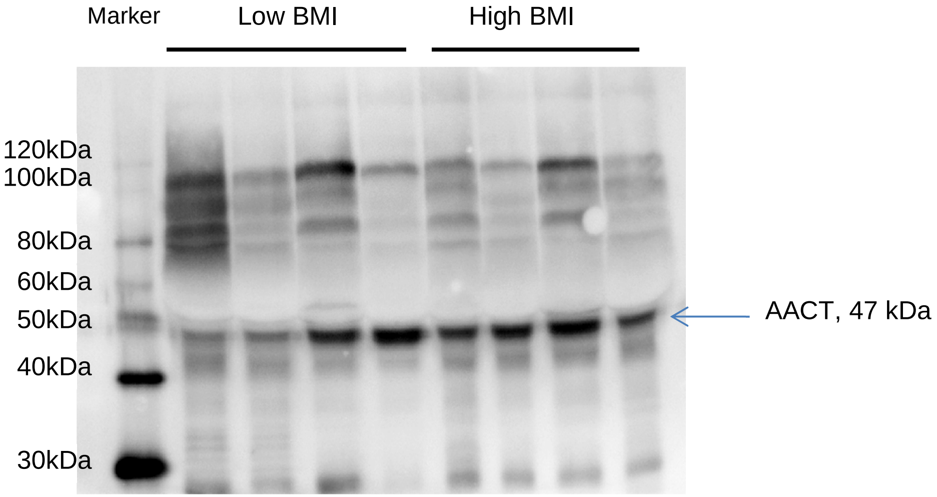

Total Stain for A), B), C) and D)

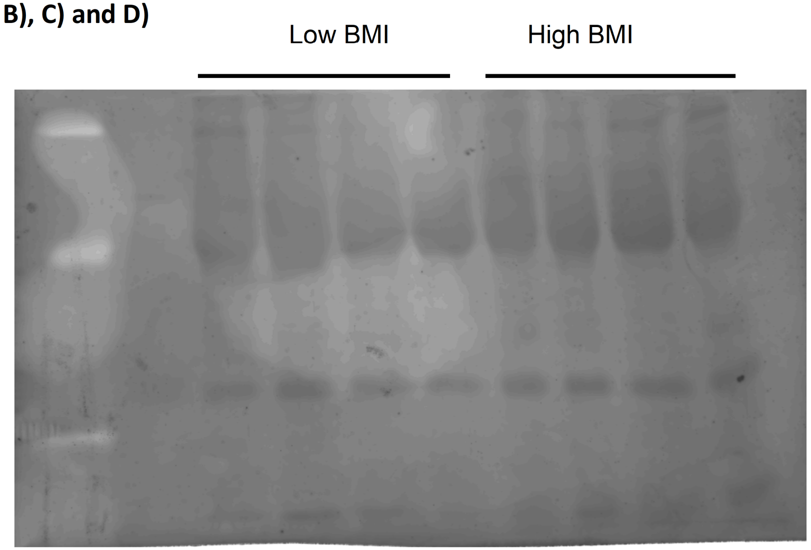

E) Apo A1.

In the upper panel the results of Apo A1 blotting is shown. In the lower panel the same image was more contrasted in order to better indicate the MW marker at 30 kDa.

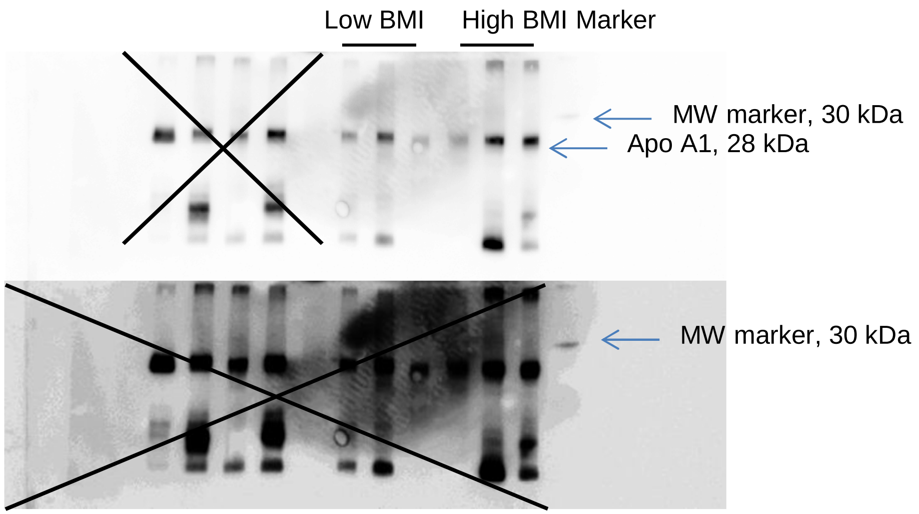

Total stain for E)

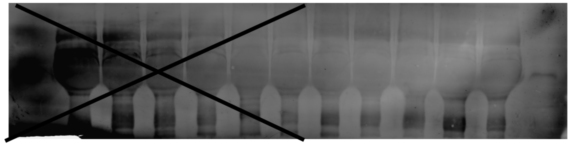

F) CC3

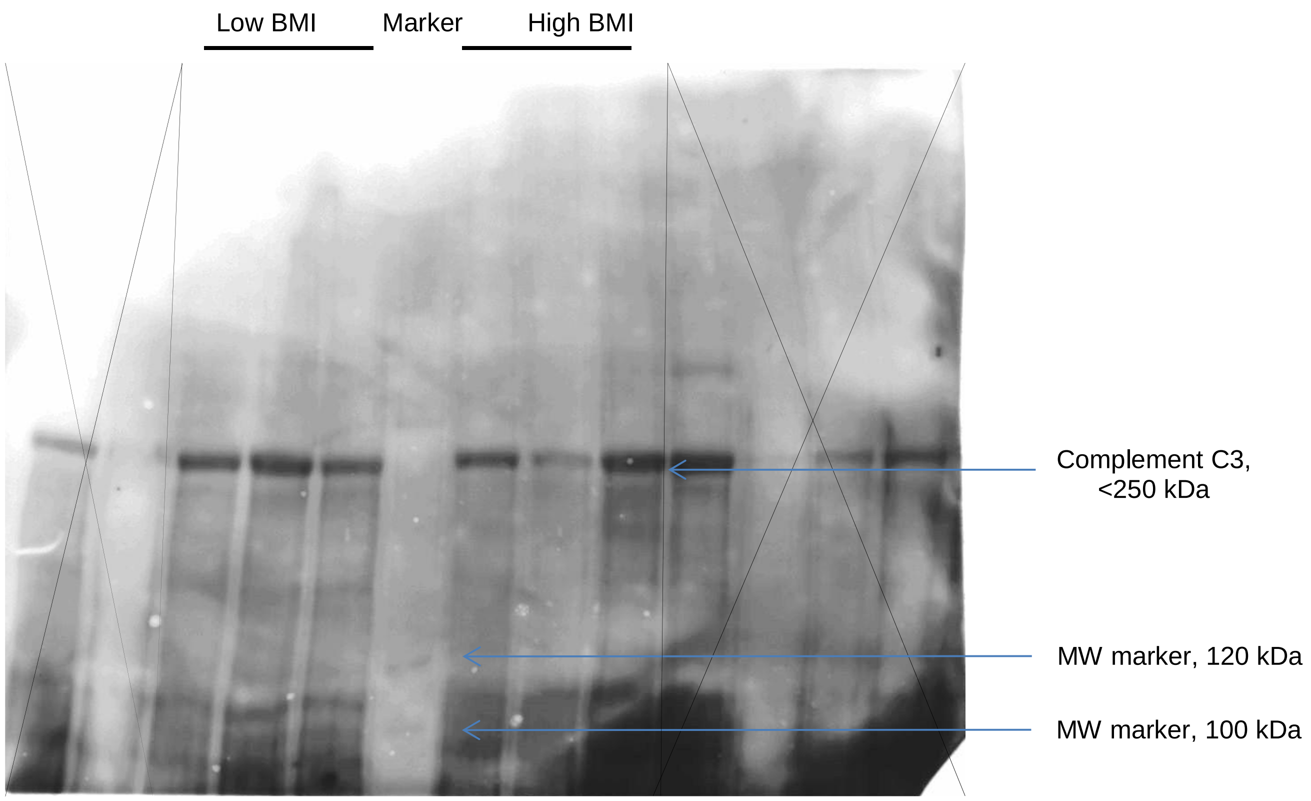

Low BMI
Marker
High BMI
Complement C3, <250 kDa
MW marker, 120 kDa
MW marker, 100 kDa

Total stain for F)

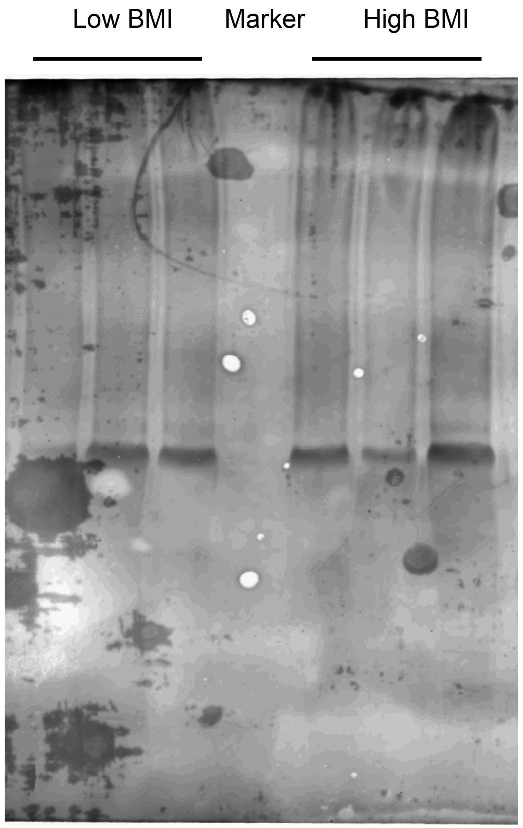

Densitometric scanning data with statistical analysis for all the intact proteins assessed by immunoblotting. Band intensities were detected using an Image Quant LAS 4000 mini imager (GE Healthcare) and assessed with the Image Quant TL 8.1 analysis software (GE Healthcare). The data were normalized against the total amount of proteins stained by Sypro Ruby and subjected to a Student's t-test.

**Apo A1**		normalised volumes				
		replicate #1	replicate #2	mean	st. dev	t-test
low BMI	subpool #1	16,2	17,9	17,7	1,5	0,006
	subpool #2	20,0	16,9			
	subpool #3	16,6	18,8			
high BMI	subpool #1	21,0	18,2	20,7	1,4	
	subpool #2	22,2	21,0			
	subpool #3	21,7	19,9			

**Apo CIII**		normalised volumes				
		replicate #1	replicate #2	mean	st. dev	t-test
low BMI	subpool #1	100,8	69,8	55,4	24,8	0,049
	subpool #2	69,0	65,6			
	subpool #3	33,4	35,3			
	subpool #4	34,7	34,6			
high BMI	subpool #1	42,3	47,8	35,6	7,6	
	subpool #2	40,3	37,0			
	subpool #3	25,6	27,6			
	subpool #4	32,1	32,0			

**Apo B100**		normalised volumes				
		replicate #1	replicate #2	mean	st. dev	t-test
low BMI	subpool #1	37,2	36,0	37,1	8,0	0,664
	subpool #2	27,7	28,1			
	subpool #3	49,1	48,2			
	subpool #4	34,6	35,8			
high BMI	subpool #1	41,7	39,7	38,6	5,9	
	subpool #2	28,5	30,1			
	subpool #3	43,4	42,0			
	subpool #4	41,5	42,3			

**LPL**		normalised volumes				
		replicate #1	replicate #2	mean	st. dev	t-test
low BMI	subpool #1	40,8	40,7	22,3	15,6	0,040
	subpool #2	37,3	20,3			
	subpool #3	21,2	6,6			
	subpool #4	7,5	3,9			
high BMI	subpool #1	32,4	33,6	45,6	24,6	
	subpool #2	105,1	32,3			
	subpool #3	41,3	47,6			
	subpool #4	36,9	36,0			

**AACT**		normalised volumes				
		replicate #1	replicate #2	mean	st. dev	t-test
low BMI	subpool #1	53,7	52,7	59,2	8,5	0,043
	subpool #2	56,6	53,1			
	subpool #3	59,0	58,9			
	subpool #4	79,0	60,3			
high BMI	subpool #1	64,7	63,8	67,1	5,3	
	subpool #2	65,5	65,4			
	subpool #3	75,4	75,5			
	subpool #4	64,1	62,2			

**CC3**		normalised volumes				
		replicate #1	replicate #2	mean	st. dev	t-test
low BMI	subpool #1	89,9	88,9	121,7	27,5	0,0007
	subpool #2	152,0	148,9			
	subpool #3	127,8	122,5			
high BMI	subpool #1	41,3	39,4	43,8	28,0	
	subpool #2	15,7	13,1			
	subpool #3	77,7	75,7			
